# Vick's Illustrated Catalogue and Floral Guide, 1878

**Published:** 1878-02

**Authors:** 


### BIBLIOGRAPHICAL.

---

*Vick's Illustrated Catalogue and Floral Guide,* 1878. Published by James Vick, Rochester, N. Y.

This enterprising florist has issued another of his annual periodicals, profusely illustrated, which must prove a valuable guide to all interested in the Flower and Vegetable Garden. Some seventy pages are devoted to a description of all of the most beautiful and popular Annuals, Climbers, Perennials, and valuable Vegetables, &c., with full directions for their proper culture and preservation.

Several years experience with the seed furnished by Mr. Vick, proves their excellence, and the reliable manner in which this business is conducted.

An Illustrated Monthly Magazine by the same publisher, is replete with valuable information, the January No. containing a beautiful and elegant colored plate representing Phlox and Pansy. Each number will contain thirty-two pages, printed on the best paper, making for the year a volume of nearly four hundred pages, and twelve colored plates. The subscription price is but $1.25 a year; or to a club of five but one dollar each. The style in which it is embellished renders this new monthly one of the most handsome periodicals published, and is a credit to its well known author, and we are assured that it will be duly appreciated, and be abundantly successful.

